# Crossing the Language Limitations

**DOI:** 10.1371/journal.pmed.0030410

**Published:** 2006-09-26

**Authors:** Zhenglun Pan, Jin Gao

We read with great interest your editorial “The Impact Factor Game” [1]. We noticed that many of the journals indexed by the Science Citation Index (SCI) pay considerable attention to impact factors and declare their figures on their journals' Web sites. We believe the game has become a most influential one in today’s scientific evaluation system. For example, some of China’s universities have adopted it as a core factor in the evaluation of the quality of research articles and recommend that students who are pursuing a doctorate publish at least one so-called “SCI-indexed paper”.

In total, 6,090 journals are indexed by SCI, most of which are published in English. However, there are many more scientific journals in the world. Over 6,300 local scientific journals are published here in China, but Chinese journals are rare in the SCI database and most of them have no impact factors.

Some may argue that the SCI database only includes the high-quality journals, but this is not necessarily the case. As a paper published in *PLoS Medicine* [2] has shown: “PubMed-indexed Chinese studies did worse than Chinese studies not indexed in PubMed in defining disease with specific criteria (17/20 [85%] versus 137/141 [97%], respectively; exact p = 0.042), and in ascertaining the eligibility of controls (13/20 [65%] versus 129/141 [92%], respectively”. The quality of an article is not determined by its language of publication.

Language accounts for much in today’s database, especially when we are searching it for evidence. Language bias should not be neglected. A language revolution could contribute to scientific progress.
